# The ecological approach to cognitive–motor dual-tasking: findings on the effects of expertise and age

**DOI:** 10.3389/fpsyg.2014.01167

**Published:** 2014-10-14

**Authors:** Sabine Schaefer

**Affiliations:** Center for Lifespan Psychology, Max Planck Institute for Human DevelopmentBerlin, Germany

**Keywords:** dual task, lifespan development, expertise, cognition, motor skills

## Abstract

The underlying assumption of studies on cognitive–motor dual-tasking is that resources are limited, and when they have to be shared between a cognitive and a motor task, performances will suffer. Resource competition should therefore be particularly pronounced in children, older adults, or people who are just acquiring a new motor skill. The current review summarizes expertise and age comparative studies that have combined a cognitive and a motor task. Expertise studies have often assessed sports performances (e.g., golf putting, soccer dribbling, rugby drills) and have shown that experts are more successful than novices to keep up their performances in dual-task situations. The review also presents age-comparative studies that have used walking (on narrow tracks or on a treadmill) as the motor task. Older adults often show higher costs than young adults, and they tend to prioritize the motor domain. These findings are discussed in relation to the ecological approach to dual-task research originally introduced by Li et al. (2005). The approach proposes to study ecologically valid dual-task situations, and always to investigate dual-task costs for both domains (cognitive and motor performance) in order to assess potential tradeoffs. In addition, task difficulties should be individually adjusted, and differential-emphasis instructions should be included in the study design.

## Introduction

Daily life consists of numerous situations in which people perform a cognitive and a motor task simultaneously. At first sight, these situations do not seem particularly challenging. In some instances, it may even help to move while thinking: Many people report that walking around the room while ruminating about a specific problem actually helps them to find a solution. However, simultaneously performing a cognitive and a motor task can become problematic if an individual's **resources** are depleted, e.g., due to old age or lack of practice. For example, a person who just learned how to ride a bike might refrain from listening to music while cycling, and older pedestrians may interrupt a conversation while crossing a busy street intersection in order to reach the other side safely. If too little attention is invested into the motor domain, the simultaneous execution of a cognitive and a motor task can result in falls or accidents with potentially severe consequences (Lundin-Olsson et al., 1997).

KEY CONCEPT 1. Resources … are not defined in a uniform manner in the literature. The concept can refer to mental effort (Wickens, 1991), or to general information processing abilities such as cognitive speed, memory span, working memory, or attention (Guttentag, 1989).

The theoretical assumption underlying many cognitive–motor dual-task studies is that resources are limited and have to be shared between the two tasks (Kahneman, 1973; Wickens, 1991). While automatized motor tasks like walking on an even surface with one's preferred speed require little attention, the situation changes if task difficulty is increased, e.g., when walking on slippery ground or over obstacles or when performing a new motor skill. Some attention is then drawn to the motor domain, and the simultaneous execution of a cognitive task may be compromised. Performance reductions from single- to dual-task conditions are often expressed as **dual-task costs**. Damos (1991) summarizes some common issues in dual-task methodology. She points out that the choice of task, practice, and whether or not participants receive feedback on their performance are crucial issues. Task difficulty is an important characteristic as well, since more resources have to be invested into difficult tasks. This review reports cognitive–motor dual-task findings from different expertise levels and different age groups.

KEY CONCEPT 2. Dual-task costs Somberg and Salthouse (1982) propose proportional dual-task costs that express performance reductions under dual-task conditions as a percentage of each individual's single-task performance. As opposed to absolute costs, proportional costs have the advantage of being comparable across age groups and task domains.

## Studies investigating the effect of expertise in cognitive-motor dual-tasking

According to the model proposed by Fitts and Posner (1967), learners acquiring a new motor skill typically go through distinct stages that differ in their demand on cognitive resources. In the cognitive stage, large parts of the movement are controlled consciously, and movement execution is slow and error-prone. The second stage, called associative stage, consists of a mixture of conscious and automatized control strategies. After extensive practice, some learners reach the autonomous stage, in which cognitive control is reduced to a minimum, and the skill can be executed efficiently, in a consistent manner, and with high movement precision. According to this model, people who are experts in a motor skill do not show performance decrements when performing the skill with a concurrent cognitive task, while novices still have to invest some attention into skill execution and perform best under single-task conditions. The performance of the concurrent cognitive task is predicted to be reduced in novices compared to single-task conditions, while experts should be able to maintain their level or performance while executing the automatized motor skill.

**Figure 1:**
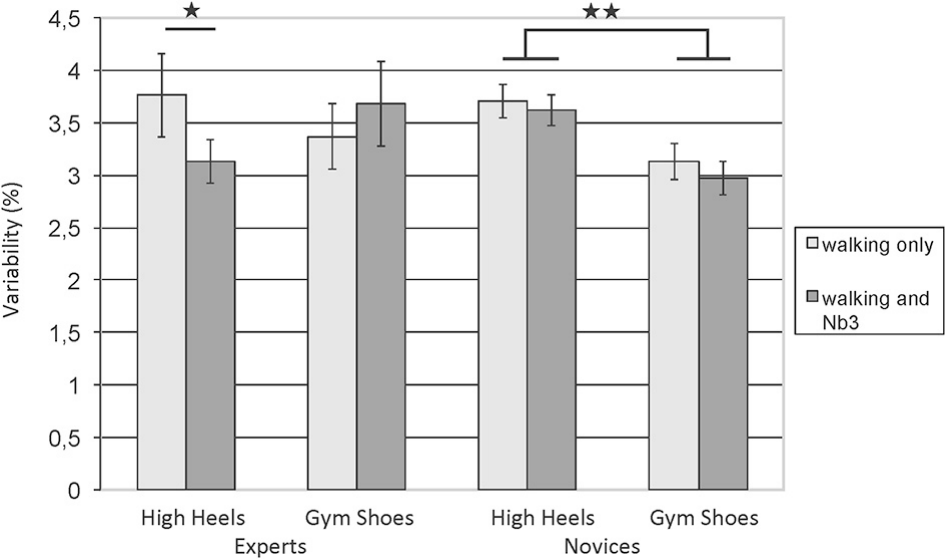
**Variability of time spent in single support (coefficient of variation)**. High-heel experts flexibly adjust their gait according to cognitive load when wearing high heels, novices show little changes in both shoe types when cognitively challenged. Error bars = SE mean. ^*^p < 0.05, ^**^p < 0.01. Figure adapted from Schaefer and Lindenberger (2013).

A study by Schaefer and Lindenberger (2013) investigated expertise related differences in high-heeled walking. The authors asked middle-aged women (40–50 years old) who either reported wearing high heels on a regular basis in everyday life (experts) and women who hardly ever wear high heels (novices), with 24 participants in each group. The motor task consisted of walking on a treadmill with one's preferred speed in gym shoes and in high heels, and the order of these sessions was counterbalanced across participants. The cognitive task taxed working memory since participants had to compare digits to digits that had been presented 3 positions earlier. Novices were expected to show performance decrements in cognition when concurrently walking in high heels, while experts were expected to keep up their cognitive performance under these conditions. However, neither group showed lower working memory performance when walking than when sitting, irrespective of shoe type. There were some gait differences between the two groups, with high-heel experts adapting their walking regularity more flexibly to shoe type and cognitive load than novices, by reducing the variability of time spent in the single-support phase of the gait cycle in high heels when cognitively challenged (see Figure 1). This indicates that high-heel expertise is associated with more flexible adjustments of movement patterns. The high-heeled shoes in the current study had been standardized across participants, with a heel height of 6 cm and an area of the heel of 4 cm^2^. Possibly high-heel expertise would have resulted in expertise-related differences in the concurrent cognitive task if the heels had been higher, or if the difficulty of the gait task had been increased by introducing uneven surfaces or gait perturbations.

**Figure 2:**
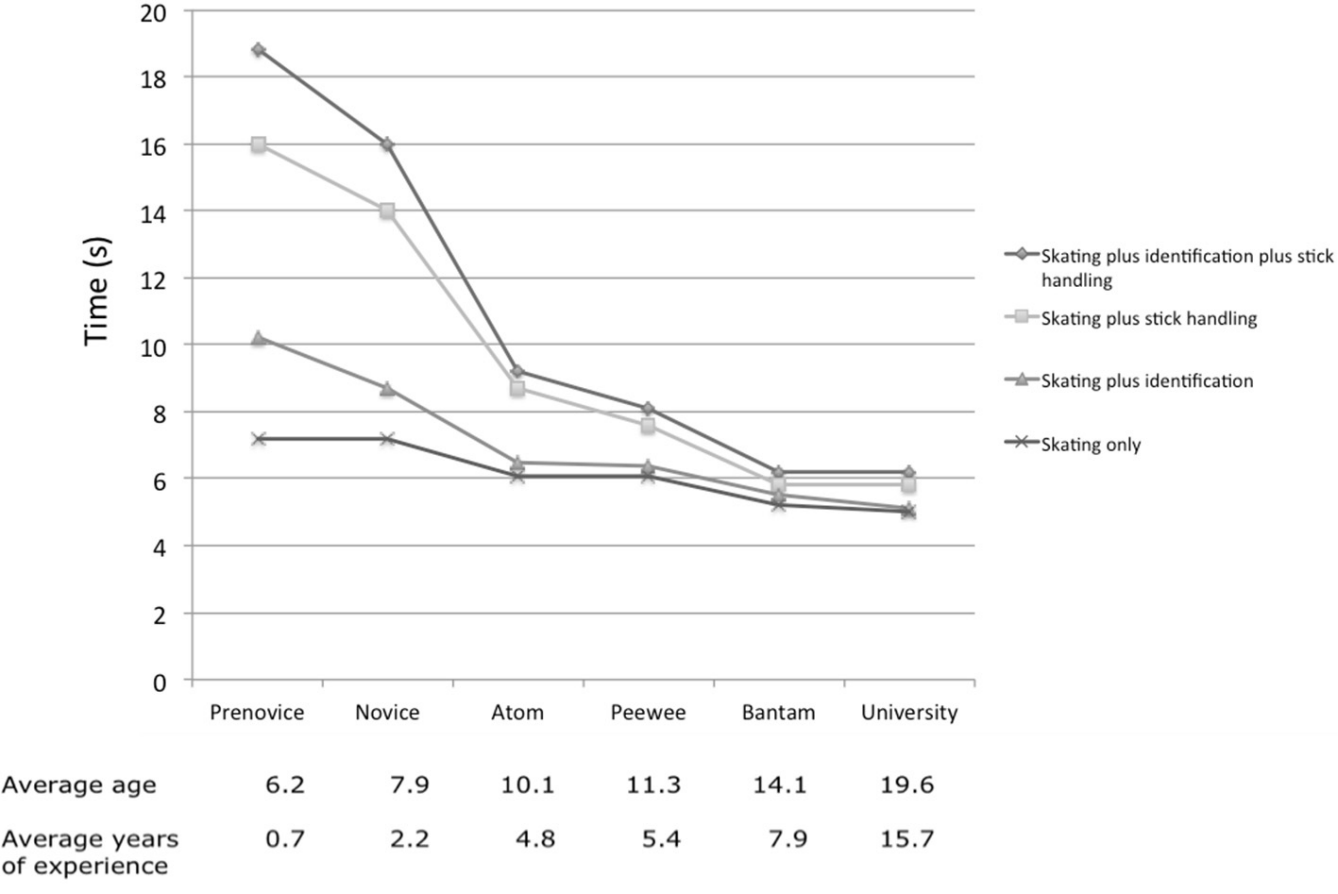
**Skating times at different expertise levels and information loads**. Figure adapted from Leavitt (1979).

Other studies on expertise differences in cognitive-motor dual-situations have focused on specific sports. An early example is the study by Leavitt (1979) who asked ice hockey players with different levels of experience, from novice to expert, to skate for speed under various condition: skating in isolation, skating while identifying geometric objects, skating plus stick handling, and skating plus stick handling while identifying objects. With increasing level of experience, speed decrements induced by the additional tasks became less pronounced. However, age and level of experience were confounded in this study, with the least experienced players being 6 years old on average, and the most experienced players being almost 20 years old (see Figure 2). Smith and Chamberlin (1992) asked novice, intermediate and expert female soccer players to run through a slalom course as fast as possible. The secondary tasks consisted of dribbling a soccer ball or of ball dribbling while identifying geometric shapes. Results showed that the players were slowed by the addition of secondary tasks, but the decrement decreased as level of expertise increased.

The studies by Leavitt (1979) and Smith and Chamberlin (1992) did not assess single-task performances in the secondary tasks, such that performance decrements in cognition could not be analyzed. To reveal potential **trade-offs** in dual-task situations, it is necessary to investigate changes in both task domains, as suggested by the ecological approach to dual-task research (Box 1). This was done in studies on rugby players. Gabbett et al. (2011) investigated high-skilled and lesser skilled rugby players performing a rugby drill under single- or dual-task conditions (while performing a verbal tone recognition task). The performance of experts was more resistant to skill decrement under dual-task conditions. Gabbett and Abernethy (2013) asked highly-skilled, intermediate-skilled and low-skilled players from three different age levels to react to virtual rugby-league specific scenarios, either in isolation or while performing the tone recognition task. In this study, there were no performance decrements in the rugby task during dual-tasking in any of the playing levels or age groups. However, in both studies participants reacted slower and committed more errors in the tone recognition task while being confronted with the rugby situations as opposed to performing the tone recognition task in isolation.

KEY CONCEPT 3. Performance trade-offs In dual-task situations, trade-offs can occur if performance decrements are more pronounced in one task domain (e.g., cognition) compared to the other (e.g., walking).

Box 1 **The model of selection, optimization and compensation (SOC) and the ecological approach to dual-task research**

The model of selection, optimization and compensation (SOC) proposed by Paul B. Baltes und Margaret M. Baltes (1990) assumes that individuals try to maximize gains and minimize losses during ontogenetic development. *Selection* refers to the selection, elaboration, and commitment to a subset of alternative life trajectories, goals, or tasks. This process prevents people from diffusing their energy into too many different domains of functioning. The model distinguishes between *elective selection* (when many different options are available, and the choice is not driven by losses) and *loss-based selection* (when the maintenance of a specific level of functioning in a given goal domain is threatened and goal hierarchies have to be modified). The second process, *optimization*, refers to situations in which resources are allocated strategically to achieve desired outcomes, for example by investing a lot of time and effort into a certain sport throughout the teenage years with the goal of taking part in the Olympics one day. The third process, *compensation*, is relevant in the management of losses and consists of the substitution of means or the use of alternative means to maintain a given level of functioning. For example, declines in sensory functioning in old age can be compensated for by the use of glasses and hearing aids.

In their *ecological approach* to cognitive-motor dual-task situations, Li et al. (2005) argue that cognitive–motor dual-task studies should …

- use laboratory tasks that are as close as possible to real-world scenarios,
- investigate the performance changes (costs) in both task domains,
- systematically vary task difficulties to challenge individuals at appropriate levels (by using adaptive testing or testing-the-limits approaches),
- include conditions in which participants are instructed to focus more strongly on one task than on the other (differential-emphasis).

By calculating costs for both domains, researchers can measure whether one task is prioritized over the other. The manipulation of task difficulties is particularly important in age-comparative studies with diverse age groups (e.g., young children vs. young adults, or very old vs. young adults). Using the same task difficulty for everybody easily results in floor or ceiling effects, and performance changes from single- to dual-task conditions may not be interpretable under these conditions. **Testing-the-limits** paradigms can be used to control for the influence of practice. The use of differential-emphasis instructions reveals whether individuals can influence their task prioritization processes.

KEY CONCEPT 4. Testing-the-limits In a testing-the-limits paradigm (Baltes, 1987), participants are systematically trained in a specific task until they reach stable performance levels. If this takes place before single- and dual-tasks are assessed, the dual-task costs are more reliable, since they are no longer influenced by practice effects.

Vuillerme and Nougier (2004) had gymnasts and a group of experts in other non-gymnastic sports perform different balance tasks (seated, bipedal stance, unipedal stance, unipedal stance on foam) while concurrently reacting to auditory stimuli. Reaction times were interpreted as an index of the attentional demand necessary for performing the postural task. Participants reacted slower when performing the more difficult postural tasks, but this effect was smaller for the gymnasts during unipedal stance, assumably because their expertise in regulating postural sway during unipedal stance enabled them to invest more cognitive resources/attention into the cognitive task.

In a study by Gray (2004), expert and novice baseball players performed a simulated baseball-batting task while judging the frequency of tones or while attending to skill execution. Novices showed degraded batting performance when concurrently judging tone frequencies, whereas experts' batting performance did not suffer. When focusing their attention on skill execution, experts showed increased batting errors and movement variability, while there was no significant effect on novices. This indicates that paying attention to the execution of an automatized motor skill can even harm performance (see also Box 2).

Box 2 **Potential explanations for different dual-task findings**

**Prioritization of posture/walking**

When performance decrements from single-to dual-task are expressed using a common metric (i.e., as proportional costs in relation to each person's single-task performance), costs can be compared between age/expertise groups and between task domains. The costs are often smaller for the motor task as compared to the cognitive task, indicating that the motor task is prioritized over cognition. Task prioritization tends to be more pronounced when people are very challenged and when the motor task involves some threat to balance/risk of falling (see also Woollacott and Shumway-Cook, 2002; Verghese et al., 2007).

**Dual-process account of sensorimotor-cognitive interactions**

Originally suggested by Huxhold et al. (2006) on the basis of findings on a balance platform, this account has also been extended to walking paradigms (Lövdén et al., 2008; Verrel et al., 2009, see also **Figure 4**). Both young and older adults profit from an external focus of attention induced by an easy concurrent cognitive task (Stoffregen et al., 1999; Swan et al., 2004). With increasing cognitive load, however, older adults' resource limitations lead to an increase in body sway or walking instability, while young adults continue to show stable levels of motor performance.

**Exercise-induced activation of resources/optimization of arousal**

There are instances in which cognitive performance can profit from exercise. While most studies in this domain have assessed the beneficial effects of exercise on cognitive tasks that are performed immediately afterwards (for reviews, see Tomporowski, 2003; Chang et al., 2012) or on long-term benefits for cognition (Colcombe and Kramer, 2003), the concurrent performance of a continuous motor task that does not tax attentional resources may lead to an optimization of arousal levels and improve cognitive performances (Kamijo, 2009; Best, 2010).

These findings were replicted with novice and experienced golfers in a study by Beilock et al. (2004). Participants performed a series of golf putts under dual-task conditions (while monitoring tones with the instruction to report the occurance of a target tone) or while focusing exclusively on skill execution. While novices performed better under skill-focus conditions, experts showed the opposite pattern.

Manipulations in the conditions under which an automatized skill is executed may also influence expertise effects. Following this logic, another study by Beilock et al. (2002a) compared novice and experienced golf putting performance in single-task (putting in isolation) and dual-task conditions (putting while performing an auditory word search task), either when using a standard putter or an arbitrarily shaped “funny putter.” With the standard putter, experienced golfers did not differ in putting accuracy from single-to dual-task conditions and had higher recognition memory for words heard while putting compared to novices. With the funny putter, which disrupted the mechanisms of skill execution, experience golfers showed decreased dual-task putting accuracy and recognition memory for secondary task words. This suggests that novel task constraints increase attention to task execution in experts. For the novices, who had as little experience with standard putters as with funny putters, performance patterns were comparable in the two conditions.

In another expertise study, Beilock et al. (2002b) obtained similar results when asking novice and expert soccer players to dribble a soccer ball through a slalom course either with their dominant or non-dominant leg, under single-task conditions or while performing a word-monitoring task. Experts profited from dual-task conditions, showing superior dribbling performance under cognitive load, when using their dominant foot. Performance for their non-dominant foot was better under skill-focus conditions. Novices performed better under skill-focus conditions regardless of foot. The authors conclude that novices and less-proficient performances of experts benefit from online attentional monitoring of step-by-step performance, while high-level skill execution is harmed.

To summarize the above findings, several studies have shown that expertise in a sensorimotor skill can be advantageous in a dual-task situation: Experts can keep up their performance in their field of expertise when faced with concurrent cognitive challenges, and they sometimes even perform better in their skill when attention is not focused on skill-execution (Beilock et al., 2002b, 2004; Gray, 2004). Novices, on the other hand, profit from skill focus conditions and usually show performance decrements in the to-be-learned skill when concurrently focusing on a cognitive task.

Not only expertise influences the attentional requirements of a motor task, but also age: Due to immaturity and lack of experience in childhood and physical declines in old adulthood, these age groups are particularly challenged when they have to perform a motor and a cognitive task concurrently. The next section therefore reviews studies comparing children or older adults to young adults.

## Age-comparative studies on cognitive-motor dual-tasking

**Figure 3:**
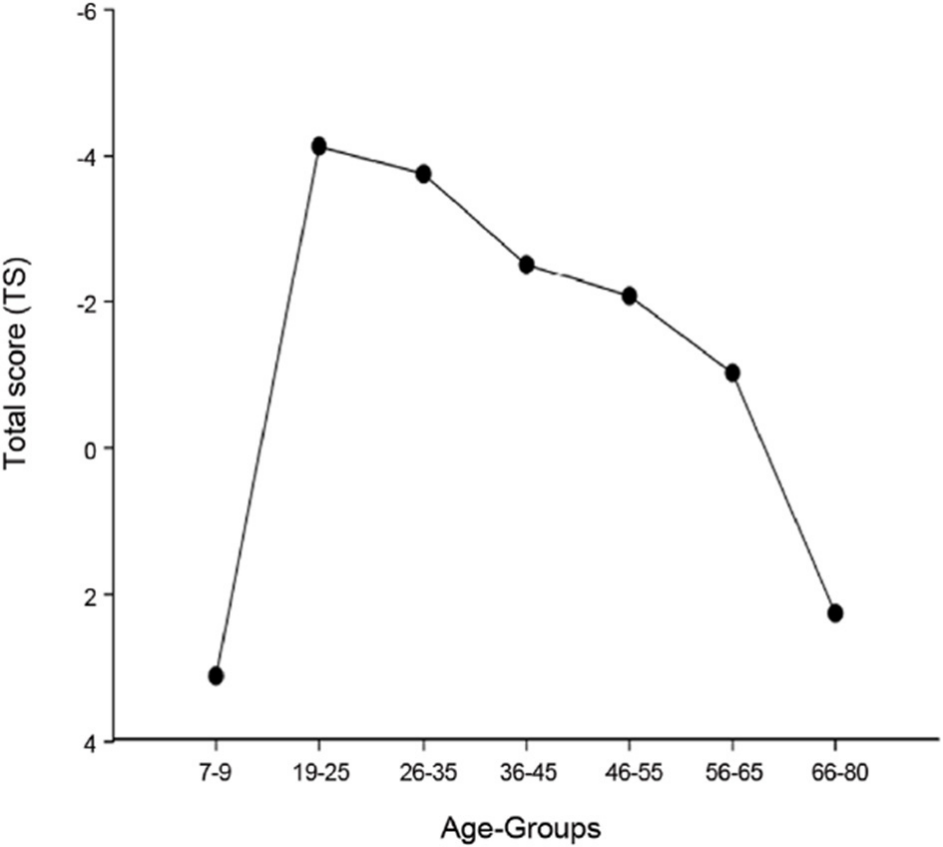
**Total scores for motor performance for different age groups, as reported by Leversen et al. (2012)**. Negative values indicate better performance.

A basic assumption of lifespan psychology is that development at every stage of life includes gains and losses (Baltes, 1987). Most performance domains show an inverted U-shaped function over the lifespan, with the highest performance level in young adulthood (for cognitive development, see Li et al., 2004; for a summary of fine- and gross-motor tasks, see Leversen et al., 2012, and Figure 3). The model of selection, optimization, and compensation (SOC), which was developed in the domain of lifespan psychology to explain how individuals regulate their development throughout the life course, can be applied to cognitive-motor dual-task research in the context of the ecological approach (Box 1). The model explains why people prioritize one task over the other in challenging situations. A central claim of the ecological approach is that dual-task costs should be investigated in both task domains, to detect potential trade-offs between cognition and motor functioning. However, many dual-task studies focus exclusively on performance changes in the motor tasks. For example, in a meta-analysis by Al-Yahya et al. (2011) on cognitive–motor interference with walking, the majority of the 66 studies did not assess single-task performances in cognition. The current report focuses on studies that have assessed both task domains, with the underlying assumption that children and older adults have fewer resources than young adults, and should therefore show larger dual-task costs. The reviewed studies are not exhaustive, but are chosen to illustrate the usefulness of an ecological approach to dual-task research with walking as the motor task. Findings from studies with patient groups (e.g., Parkinson's disease, very frail older adults, children with ADHD) have not been included. For additional reviews and meta-analyses, the interested reader is referred to Al-Yahya et al. (2011) and Beurskens and Bock (2012) who focus on cognitive-motor interference while walking, to Schaefer and Schumacher (2011) reviewing cognitive-motor interdependencies in old age, and to Woollacott and Shumway-Cook (2002) reviewing studies on attention in the control of posture and gait. The studies are grouped by walking task, contrasting treadmill walking to walking on narrow tracks.

### Walking on narrow tracks

One of the first studies in the context of the ecological approach was conducted by Lindenberger et al. (2000). They asked young (20–30 years), middle-aged (40–50), and older adults (60–70) to walk as quickly and accurately as possible on two narrow tracks that differed in task complexity. Both tracks were only 19 cm wide. Participants had to pay some attention to the walking task to avoid outstepping the tracks' boundaries. The more difficult track additionally contained several turning points of different angles. Walking performance was measured by walking speed and accuracy. The cognitive task consisted of memorizing word lists, for which participants had been trained to criterion in a mnemonic technique. Participants encoded the word lists while sitting, standing, or walking on either track. Walking performance was assessed with and without concurrent memory encoding. The main finding of this study was that dual-task costs increased with age in both domains. Relative to the young adults, the effect size of the overall increase in proportional dual-task costs was 0.98 standard deviation units for middle-aged adults and 1.47 standard deviation units for old adults. In addition, when directly comparing the dual-task costs across the two task domains on the easier track, younger adults showed higher costs in walking than in cognition, middle-aged adults showed comparable costs across the two domains, and older adults showed higher costs in cognition, indicating that they prioritize the motor domain. On the more difficult track, however, dual-task costs were larger in the memory domain as compared to the walking task for all three age groups. This indicates that resource requirements and task difficulty both influence task prioritization processes. Keeping up one's motor performance under demanding conditions can be considered adaptive, since it may protect people from falls and their potentially severe consequences (Lundin-Olsson et al., 1997).

A study by Li et al. (2001) asked young and older adults to walk on a narrow track while concurrently encoding word lists. The track in this study corresponded to the easy track of the study by Lindenberger et al. (2000). In some conditions, walking difficulty was increased by asking participants to step over obstacles. For the memory task, participants were instructed in a mnemonic technique, and task difficulty was manipulated by shortening the inter-stimulus interval between to-be-encoded words. Before the dual-task phase started, participants received extensive training in the two tasks, and they were also accustomed to using external aids (holding on to a handrail while walking, or prolonging the inter-stimulus interval via button presses in the encoding phase of the memory tasks). Age differences in dual-task costs were larger for memory than for walking, again suggesting that older adults prioritized walking over memory. This can be considered an incidence of loss-based selection according to the SOC-model (see Box 1). In the trials in which external aids could be used, older adults optimized walking by holding on to the handrail, while younger adults optimized memory performance. Besides replicating the finding that older adults prioritize motor over cognitive performance, the results also emphasize the need to assess costs as well as benefits when designing assistive devices for older adults (Lindenberger et al., 2008).

Older adults show a strong tendency to prioritize their motor functioning in demanding dual-task situations. However, it remains an open question whether children, who also have fewer resources available than young adults, will show a similar tendency. An alternative prediction is that risk-tasking more adaptive in childhood, since many new motor skills cannot be acquired without taking risks.

To investigate such tendencies in children, Krampe et al. (2011) asked 9-year-olds, 11-year-olds, young adults, and older adults to walk on the narrow track while concurrently performing a word fluency task (e.g., to name as many four-legged animals as possible in a given time). Task difficulties of the categories used in the word fluency task had been piloted for each age group and were counterbalanced across single-and dual-task trials. Distances walked and number of category exemplars retrieved showed an inverted U-shaped function with age, with children and older adults performing worse than young adults. Proportional dual-task costs in walking (expressing performance decrements as a percentage of each individual's single-task performance) also showed higher costs for children and older adults than for young adults. Only 9-year-olds showed significant costs in the cognitive task, indicating that task prioritization in favor of the motor task could not be detected in this study. This may be due to the rather low difficulty of the motor task: Walking on a narrow track is easier than walking on a track with numerous turning points or obstacles. This demonstrates older adults' and children's abilities to accommodate their resource allocation to the ecological constraints of the setting.

### Treadmill walking

Walking on a narrow track poses some threat to balance, since the feet have to be placed within the boundaries of the track. Many cognitive-motor dual-task studies used treadmill walking instead. After familiarization on the treadmill, people show similar walking patterns as in walking on the ground for most gait parameters (Schellenbach et al., 2010). Nevertheless, being able to stabilize oneself while walking on a treadmill by holding on to a handrail helped older men in a concurrent spatial navigation task, while it did not influence navigation performance in younger men (Lövdén et al., 2005). Gait variability is often used as the dependent measure for treadmill walking, and it can either be expressed as the coefficient of variation of gait parameters such as stride length, stride time, cadence, and velocity (Hausdorff, 2005), or as the residual components of whole-body motion in principal component analysis (PCA; Verrel et al., 2009). A more variable gait is often interpreted as being more instable, which may lead to an increased risk of falls, especially in older adults (Springer et al., 2006; Callisaya et al., 2009; Yogev-Seligmann et al., 2013).

**Figure 4:**
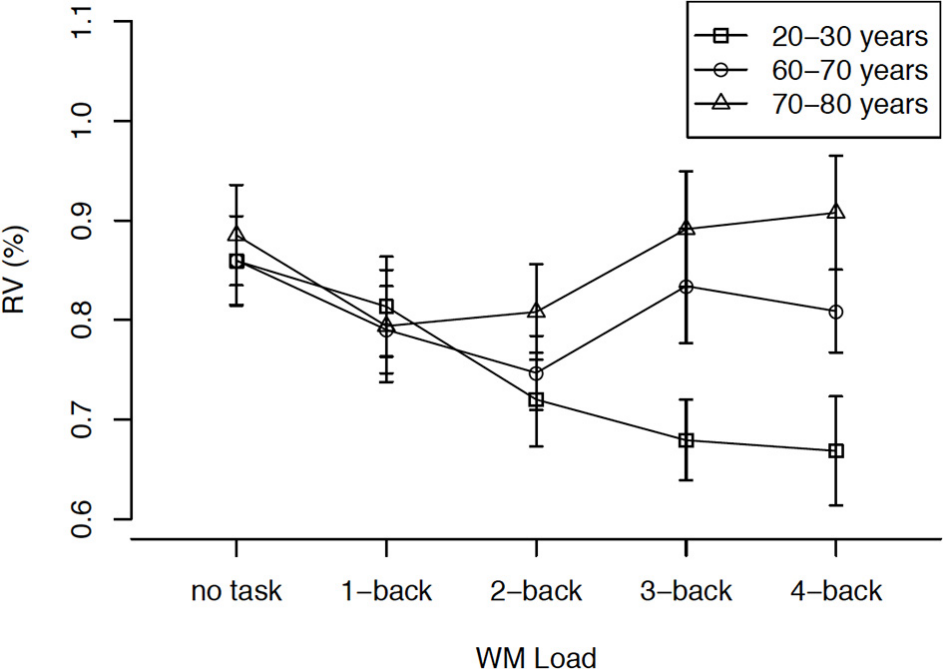
**Residual variance (RV) as a function of age group and working memory (WM) load after extracting the first six principal components in a whole-body motion analysis of walking data**. Error bars = SE mean. Figure adapted from Verrel et al. (2009).

Lövdén et al. (2008) asked 32 younger (aged 20–30 years) and 32 older adults (60–70 years) to walk on a treadmill with their preferred speed. The cognitive task taxed working memory since participants had to compare digits to digits that had been presented *n* positions earlier (n-back). Task difficulty was varied from 1-back to 4-back for each individual. There were no cognitive dual-task costs in this study: Young and older adults showed comparable performance levels when working on the n-back task while sitting on a chair or walking on the treadmill. Stride-to-stride variability was lower when participants performed an easy working-memory task (1-back) than when they walked without a cognitive task, suggesting that automatized motor tasks are performed more smoothly if attention is focused on something else (instead of using an internal focus of attention). When task difficulty was increased by moving from 1- to 4-back, younger adults further decreased their stride-to-stride variability, while older adults did not. Thus, increasing the cognitive demand of the concurrent task may no longer be advantageous for older adults. Verrel et al. (2009) extended this paradigm by adding a group of even older adults (aged 70–80 years). The authors used the residual variance of a PCA as an index of gait stability. The oldest participants showed an increase of residual variance in the highest load conditions, indicating a less stable gait when cognitively challenged. While the effects of internal vs. external focus of attention generalize across the three age groups, the effects of resource competition with increasing cognitive–task difficulty are age-specific, influencing those participants with fewer resources (i.e., the oldest) more strongly. Figure 4 presents the residual variance after extraction of the first six principal components.

Can these findings be generalized to children? A study by Schaefer et al. (2010) investigated how 9-year-old children and young adults performed the n-back task (difficulty levels 1- to 4-back) while walking on a treadmill. Two different walking conditions were administered in this study: Walking at one's preferred speed or walking at a fixed speed of 2.5 km/h. As in the studies with older adults, stride-length and stride-time variability decreased with growing cognitive load in young adults, whereas children showed an increase in variability when cognitive load was very high. Interestingly, participants in both age groups *improved* their cognitive performance when walking on the treadmill at their preferred speed as opposed to sitting, or walking at their non-preferred speed. This finding cannot be explained by resource limitations. Instead, walking at their preferred speed may have optimized arousal levels in the participants. Walking at 2.5 km/h, which was slower than the preferred speed for each participant, possibly demanded some attention again, such that participants were no longer able to cognitively profit from the exercise.

## Conclusions and practical implications

The above summary of various cognitive–motor dual-task studies shows that there is no universal explanation that can predict the pattern of dual-task costs in a particular situation. Box 2 presents an overview of the accounts that have been used to explain the findings. Cognitive–motor dual-task situations seem to be influenced by a variety of factors, such as task difficulty, motivational preference, arousal level, internal vs. external focus of attention, and postural threat.

The ecological approach to dual-task research argues that *task difficulty* should be individually adjusted to avoid potential floor or ceiling effects. Although this procedure is well-established in developmental research (for examples, see Li et al., 2001; Brehmer et al., 2007; Schaefer et al., 2008) and it may be the only way to calculate reliable dual-task costs for each individual, the procedure possibly reduces ecological validity: In the real world, people of all ages and ability levels are faced with the same tasks. Stairs are equally high for children, young, and older adults. Escalators do not reduce their speed for seniors, and subway announcements have a certain loudness and clarity. People have to adapt to these challenges, and some may not be able to deal with them at all. To measure such a complete “break-down” of performance in demanding situations could be at least as informative as the results of studies that invest a lot of time and effort into the individual calibration of task difficulties. If actual falls are too risky, laboratory environments can work with virtual world scenarios to assess situations in which falls or accidents would occur in the real world (for age-comparative work with a virtual street crossing paradigm, see Neider et al., 2011).

From a lifespan developmental point of view, it is interesting to examine the question how people adapt to changes in their resources. The SOC theory presented in Box 1 (Baltes and Baltes, 1990) suggests that they use different strategies to optimize developmental outcomes. In a cognitive–motor dual-task situation, risk-taking may be particularly rewarding for children, allowing them to experience new challenges and to improve their skills in a variety of settings (Morrongiello and Dawber, 2004; Christensen and Mikkelsen, 2008). However, accidents are prevalent in childhood and can have severe consequences. According to WHO statistics (WHO, 2008), they account for up to 25% of emergency department visits worldwide. In old age, consequences of falls are often even more dramatic, potentially leading to injuries, disabilities, or even death. Although advising older adults to be extremely careful in challenging motor situations seems like a good strategy, introducing exercises for strength and balance that prevent falls are just as important. Older adults may lose their ability to react adequately if potentially difficult situations are avoided entirely. Successfully negotiating the need for practice and experience vs. safety considerations are the big challenges in response to cognitive–motor dual tasks throughout the lifespan.

To conclude, expertise as well as age can influence performance patterns in cognitive-motor dual-task situations. Experts reduce their motor performances to a lesser extent than novices under cognitive load, and they sometimes even profit from a concurrent cognitive task. This indicates that skilled sportsmen may perform better when not focusing their attention on skill execution, and this knowledge can be useful in training and competitions. Older adults have larger costs in cognitive-motor dual task situations than their younger counterparts, and tend to prioritize the motor domain, whereas children do not always show performance decrements. For a more thorough understanding of the mechanisms underlying different patterns of findings, future research should consider the assumptions of the ecological approach to dual-task research (Box 1), that is, pay attention to factors like choice of task, task difficulty manipulations, and differential-emphasis instructions.

### Conflict of interest statement

The author declares that the research was conducted in the absence of any commercial or financial relationships that could be construed as a potential conflict of interest.
